# Harnessing epigenome modifications for better crops

**DOI:** 10.1093/jxb/erw143

**Published:** 2016-05-07

**Authors:** James Giovannoni

**Affiliations:** US Department of Agriculture Robert W. Holley Center and Boyce Thompson Institute, Tower Road, Cornell University campus, Ithaca, NY 14853, USA

**Keywords:** Auxin, DNA methylation, epigenome, genetic diversity, paramutation, *sulfurea*, tomato.

**Chemical DNA modifications such as methylation influence translation of the DNA code to specific genetic outcomes. While such modifications can be heritable, others are transient, and their overall contribution to plant genetic diversity remains intriguing but uncertain. In this issue, Gouil and colleagues (pages 2655–2664) characterize the epigenome phenomenon termed ‘paramutation’ underlying a tomato photosynthesis-related gene defect, and in doing so expand our understanding of how epigenome modifications are conferred with exciting implications for crop improvement.**

It has long been recognized that DNA harbors information of richer texture and fidelity than the DNA sequence alone. Just as recorded texts contain information while volume, emphasis and accent contribute to the ultimate meaning conveyed by language, DNA sequence presents the genetic code while epigenome modifications influence specific conversion of this code into phenotypic traits. Epigenome contributions to genetic diversity and outcomes are gaining in appreciation as genomes and their modifications become ever more readily characterized and cataloged.

As examples, whole genome sequencing and genomic DNA–protein interaction studies have revealed that many genomes, including those of important crops, include large repertoires of DNA- and RNA-derived mobile genetic elements rendered silent via chemical modifications, including cytosine methylation and methylation or acetylation of DNA-associated histone proteins (Cui and Cao, 2014). Also heritable epigenome reprogramming is critical in plant embryo development (Schmitz and Ecker, 2012), while developmental epigenome changes contribute to fruit maturation and ripening (Zhong *et al.*, 2013), with additional modifications acquired in response to stress conditions. Recent evidence suggests the existence of molecular mechanisms in place to erase these induced modifications prior to meiotic transfer (Iwasaki and Paszkowski, 2014). A number of epi-alleles (stable genetic variants in DNA methylation patterns, but not DNA sequence) have been reported (Weigel and Colot, 2012), confirming that some heritable genetic diversity is anchored in the epigenome.

The realization that a number of genetic diseases are traceable to the epigenome, including some cancers (Timp and Feinberg, 2013), has accelerated interest and inquiry into mechanisms of epigenome modification and resulting manifestations that can contribute to illness. Similarly, research in plants is spurred on by the knowledge that a clearer mechanistic picture of epigenome mechanisms will facilitate our understanding of their relative contributions to crop genetic diversity and open doors to their use for crop improvement.

## Paramutation facilitates genetic diversity via epigenome modification

Paramutation has been studied extensively in maize, though examples have been reported in additional species, including tomato, at the *sulfurea* (*sulf*) locus. The phenomenon was first described as an apparent exception to Mendel’s First Law of allelic segregation (Brink, 1956) where an inactive allele of the maize gene *r1* was observed to convert, or paramutate, an active (paramutable) *R1* allele to the inactive form. The resulting paramutated allele was both heritable and capable of conferring the same effect on other active alleles when transferred via sexual hybridization. While the underlying DNA has been shown to encode tandemly repeated copies of an HLH transcription factor regulating anthocyanin biosynthesis (Dooner *et al.*, 1991), better characterized examples of maize paramutation at the *b1* and *pl1* loci (Hollick, 2012) indicate that small interfering RNAs (siRNAs) are associated with DNA methylation changes responsible for these paramutagenic phenomena (Box 1).

**Box 1. Endogenous paramutation and targeted gene silencing**

**Figure:**
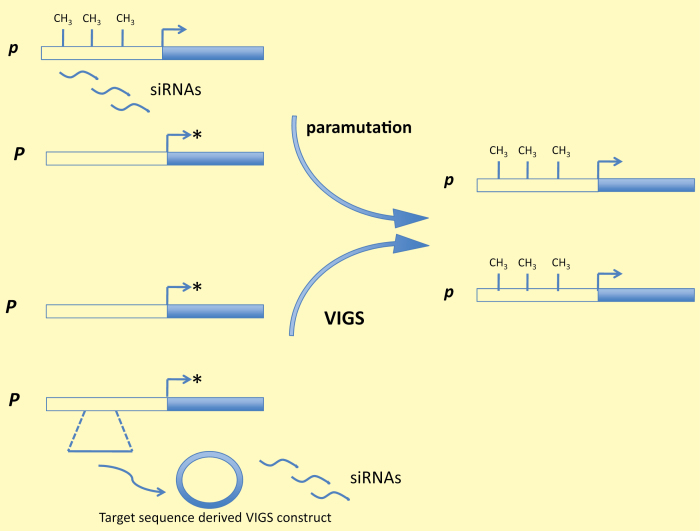
Endogenous paramutation and targeted gene silencing – e.g. virus-induced gene silencing (VIGS) – confer siRNA-mediated DNA methylation changes on susceptible loci. In this example the hypomethylated and transcriptionally active (*) *P* allele is converted to the hypomethylated and inactive *p* allele by either mechanism. A range of genetic outcomes could be achieved through differential methylation highlighting the potential for targeted epigenome and trait engineering.

## Tomato as a model for epigenome analysis

While maize is among the most important staple crops and a model for plant genetics, the cultivated tomato (*Solanum lycopersicum*) offers a number of advantages for experimentation. These include a smaller and less repetitive sequenced genome; the ability to create stable inbred and true-breeding lines; efficient means of stable and transient DNA transformation; and a large collection of characterized genetic mutations, variants and inter-fertile wild species facilitating genetic analyses (The Tomato Genome Consortium, 2012 and references therein).

Gouil *et al.* (2016) took advantage of these features in characterizing the paramutagenic tomato *sulf* locus, which manifests as sectors of chlorosis (i.e. yellowing, thus the *sulfurea* designation) in *sulf*/+ leaves where paramutation has rendered the corresponding somatic genotype *sulf*/*sulf*. Genetic segregation in a population derived from a cross with the wild progenitor (*Solanum pimpinellifolium*) of the cultivated tomato confirmed prior localization of *sulf* to a region of chromosome 2. The tomato genome sequence allowed identification of a collection of linked gene candidates and further narrowed this group to a subset with differential gene expression via whole genome transcriptome analysis of normal and mutant genotypes.

The most downregulated gene was *SLTAB2*, whose Arabidopsis ortholog was previously linked to translation of mRNAs encoding photosystem proteins (Barneche *et al.*, 2006). In the absence of proper photosystem assembly, chloroplast membrane structure and photosynthetic capacity is dramatically compromised leading to the yellowing phenotype characteristic of both *sulf* and a corresponding Arabidopsis T-DNA mutant. Promoter methylation profiles revealed regions of differential methylation in *sulf* versus wild-type *SLTAB2*. Especially noteworthy was a hypermethylated region that encompassed the transcription start site, likely contributing to downregulation of *SLTAB2* in *sulf* genotypes and termed DMR1 (Differentially Methylated Region 1). This region was also associated with elevated levels of 23–24-nucleotide siRNAs associated with RNA-mediated DNA methylation. DNA sequence variation between the cultivated and wild species alleles of the *sulf* locus further allowed monitoring of paramutation in the segregating F1 population where the wild species allele also became hypermethylated at DMR1 in the presence of the *sulf* allele.

## Epigenome modification as a tool for crop improvement

Paramutation and its consequences were not only directly monitored in the segregating population via promoter methylation, but were elegantly reconstituted through transient expression of DMR1 sequences in wild-type tomato plants. Expression of non-transcribed sequences has been linked to RNA-mediated DNA methylation of the corresponding genomic sequences (Box 1) with consequences for expression of linked genes (Bond and Baulcombe, 2015). Indeed expression of DMR1 sequences in normal tomato plants resulted in elevated levels of both DMR1 promoter methylation and corresponding 23–24-nucleotide siRNAs, in addition to leaf chlorosis analogous to the *sulf* mutation.

These results not only provide confirmation of the DNA sequence target and nature of epigenome changes conferring the *sulf* phenotype, but also demonstrate the potential for creating novel genetic variants through targeted epigenome modification. Such modifications would be expected to yield a range of phenotypes corresponding to the degree of methylation modification and with applications to specific and desired phenotypic outcomes. As an example, one might imagine a collection of alleles that confer increased fruit ripening delays tailored to localized post-harvest storage needs. Alternatively, a range of expression levels from a locus conferring salt tolerance, but at a cost to yield, could be tailored to localized soil conditions so as to facilitate acceptable crop performance while minimizing adverse side effects.

## Contribution of the epigenome to natural genetic diversity

The dramatic effect of epigenome state on phenotype highlighted by the tomato *sulf* allele begs the question of the role of epigenome variation in available germplasm that is deployable for crop improvement. Many domesticated species, including tomato, are derived from a relatively small slice of the pie of available genetic diversity, yet retain considerable phenotypic variability. In inbred species, where tomato is again an example, DNA sequence diversity may be limited and epigenome diversity may play a larger role in trait variability. Indeed tomato breeders have long noted that while phenotypic diversity abounds in germplasm collections, DNA sequence-based molecular markers facilitating selection of desired traits are usually difficult to identify. Further investigations into epigenomes of model crop systems such as tomato will reveal the contributions of the epigenome to genetic diversity, and should highlight methods to select for and possibly manipulate phenotypic range and eventually culminate in the development of targeted methods for tailoring epigenomes toward enhancement of desired crop traits. The detailed characterization of the *sulf* gene by Gouil *et al.* (2016) suggests these aims are achievable.

## References

[CIT0001] Barneche F Winter V Crèvecoeur M Rochaix J-D 2006 ATAB2 is a novel factor in the signalling pathway of light-controlled synthesis of photosystem proteins. The EMBO Journal 25, 5907–5918. 17139246 10.1038/sj.emboj.7601472 PMC1698907

[CIT0002] Bond D Baulcombe DC 2015 Epigenetic transitions leading to heritable, RNA mediated de novo silencing in *Arabidopsis thaliana* . Proceedings of the National Academy of Sciences, USA 112, 917–922. 10.1073/pnas.1413053112 PMC4311854 25561534

[CIT0003] Brink R 1956 A genetic change associated with the R locus in maize which is directed and potentially reversible. Genetics 41, 872–889. 17247669 10.1093/genetics/41.6.872 PMC1224369

[CIT0004] Cui X Cao X 2014 Epigenetic regulation and functional exaptation of transposable elements in higher plants. Current Opinion in Plant Biology 21, 83–88. 25061895 10.1016/j.pbi.2014.07.001

[CIT0005] Dooner H Robbins T Jorgensen R 1991 Genetic and developmental control of anthocyanin biosynthesis. Annual Review of Genetics 25, 173–199. 10.1146/annurev.ge.25.120191.001133 1839877

[CIT0006] Gouil Q Novák O Baulcombe D 2016 *SLTAB2* is the paramutated *SULFUREA* locus in tomato. Journal of Experimental Botany 67, 2655–2664. 10.1093/jxb/erw096 PMC4861014 26957563

[CIT0007] Hollick JB 2012 Paramutation: a trans-homolog interaction affecting heritable gene regulation. Current Opinion in Plant Biology 15, 536–543. 23017240 10.1016/j.pbi.2012.09.003

[CIT0008] Iwasaki M Paszkowski J 2014 Identification of genes preventing transgenerational transmission of stress-induced epigenetic states. Proceedings of the National Academy of Sciences, USA 111, 8547–8552. 10.1073/pnas.1402275111 PMC4060648 24912148

[CIT0009] Schmitz R Ecker J 2012 Epigenetic and epigenomic variation in *Arabidopsis thaliana* . Trends in Plant Science 17, 149–154. 22342533 10.1016/j.tplants.2012.01.001 PMC3645451

[CIT0010] **The Tomato Genome Consortium** 2012 The tomato genome sequence provides insights into fleshy fruit evolution. Nature 485, 635–641. 22660326 10.1038/nature11119 PMC3378239

[CIT0011] Timp W Feinberg A 2013 Cancer as a dysregulated epigenome allowing cellular growth advantage at the expense of the host. Nature Reviews Cancer 13, 497–510. 10.1038/nrc3486 PMC4636434 23760024

[CIT0012] Weigel D Colot V 2012 Epialleles in plant evolution. Genome Biology 13, 249. 23058244 10.1186/gb-2012-13-10-249 PMC3491404

[CIT0013] Zhong S Fei Z Chen Y 2013 Single-base resolution methylomes of tomato fruit development reveal epigenome modifications associated with ripening. Nature Biotechnology 31, 154–159. 10.1038/nbt.2462 23354102

